# Poisoning by Antifebrin

**Published:** 1890-08-09

**Authors:** 


### POISONING BY ANTIFEBRIN.

Dr. Vierhuff, of Petersburg, reports a case which very nearly proved fatal. A healthy young lady, awaking with a severe headache, took [a teaspoonful of antifebrin about seven a.m., and relief not following immediately she repeated the dose ten minutes after the first. On her husband observing that he thought such doses were dangerous, she drank a glass of milk and a solution of alum, with the view of producing vomiting, but without any result.

Soon she felt vertigo, tinnitus aurium, throbbing of the temporal arteries, dull headache, and great weakness. Her complexion, according to her husband's account, assumed a blue-green hue. When Dr. V. arrived about eleven a.m., or four hours after, her face was sallow and livid, the lips, finger and toenails blue, the surface of the whole body of an extraordinary pallor, and the pupils contracted, the heart sounds weak, the pulse 84, temp. 35·4 deg. C. (97·5 deg. F.)

He ordered her a teaspoonful of Carlsbad salts as an aperient, and for a stimulant cold brandy and water in default of wine.

In half-an-hour collapse suddenly set in, the heart's action became irregular and intermittent, the pulse almost imperceptible and not to be counted, respiration shallow, and consciousness was completely lost.

Dr. V. then ordered vigorous stimulation of the skin by brushing the soles of the feet, rubbing vinegar on the face, and dashing cold water on the surface of the body. Subcutaneous injections of two grams of a mixture of ether and camphorated oil were repeated at intervals, when consciousness gradually returned, but was soon lost again. Then violent vomiting by mouth and nose set in, with death-like faintness, from which she could only with difficulty be partially roused. Collapse returned several times, and she lay in this condition, almost as if dead, for between three and four hours, at the end of which she improved a little. Several hours later, another attack of syncope coming on, an intravenous injection of a solution of common salt was administered by means of an irrigator, with which a Pravaz' needle was connected by small india rubber tubing, so gently that it took two hours to introduce half-a-pint of the solution, ether injections being performed meanwhile. When the saline injection was concluded she had so far recovered that further stimulation was not necessary. In fourteen hours, or about nine p.m., she was out of all immediate danger as regarded her life, but she long remained anæmic and weak. More than a week afterwards she was still unable to walk about the room without assistance, and for some time longer she complained of pains in all her limbs.

